# Characterising Alternative Diagnoses to Psoriatic Arthritis in a Rheumatology‐Dermatology Combined Clinic

**DOI:** 10.1111/ajd.14585

**Published:** 2025-08-21

**Authors:** Ugochukwu Kingsley Odega, Wilson Omesiete, Adam Carlson, R. Hal Flowers

**Affiliations:** ^1^ University of Virginia School of Medicine Charlottesville Virginia USA; ^2^ Department of Dermatology University of Virginia Health System Charlottesville Virginia USA; ^3^ Department of Rheumatology University of Virginia Health System Charlottesville Virginia USA

**Keywords:** clinical dermatology, general dermatology, inflammatory arthritis, psoriasis, psoriatic arthritis, rheumatology

## Abstract

**Background:**

Psoriatic arthritis (PsA) is a chronic inflammatory arthritis involving axial and peripheral joints and tendons that affects a subset of patients with psoriasis (PsO). PsA can be a debilitating disease and warrants prompt rheumatologic evaluation and management. The diagnosis of PsA can be challenging for the practising dermatologist as there is often an overlap in the symptoms of PsA and non‐inflammatory musculoskeletal conditions such as osteoarthritis, tendonitis and myofascial pain.

**Aims:**

The primary aim of the study is to examine psoriasis patients seen at our tertiary institution's combined rheum‐derm clinic for a concern for PsA, specifically examining the revised diagnosis and the joint symptom similarities to psoriatic arthritis.

**Methods and Material:**

We performed a retrospective chart review of patients referred to the rheumatology‐dermatology clinic (RDC) at our institution between November 2019 and March 2022. Our review included 242 patients, of which 34 (14%) were psoriasis patients specifically referred due to suspicion for PsA. Each patient underwent a comprehensive rheumatic evaluation, including history, physical examination, laboratory tests and imaging as needed.

**Results:**

Fourteen (41.2%) of the 34 patients referred for suspected PsA were diagnosed with non‐inflammatory musculoskeletal conditions, primarily mechanical joint pain. Stiffness and/or swelling were significantly more common among patients with confirmed PsA.

**Conclusions:**

These findings underscore the importance of thorough evaluation of musculoskeletal symptoms, particularly stiffness and swelling, in patients with psoriasis. Improving education on distinguishing non‐inflammatory musculoskeletal conditions may enhance diagnostic accuracy and optimise referral practices.

## 1 Introduction

Psoriatic arthritis (PsA) is a chronic inflammatory condition of the joints and skin with a prevalence of 0.3%–2% in the general population and 6%–42% in patients with psoriasis [1, 2, 3, 4]. This potentially debilitating disease warrants prompt rheumatologic evaluation [5, 6]. No specific laboratory tests or x‐ray findings diagnose PsA, so a combination of skin and joint signs and symptoms is often used to establish a diagnosis [1]. The diagnosis of PsA in psoriasis patients can at times be challenging [7, 8]. Interdisciplinary rheumatology‐dermatology clinics (RDCs) bring together multiple specialists to provide more holistic care for psoriatic patients. This collaborative approach has been shown to enhance patient outcomes and satisfaction by facilitating earlier and more accurate PsA diagnoses and interventions [7, 9, 10].

Prior to referral to the rheumatology‐dermatology clinic (RDC), patients with psoriasis are frequently diagnosed by the referring physician as having psoriatic arthritis or musculoskeletal pain. However, during their RDC consultations, a number of these patients may have their diagnoses revised. A retrospective study by Valez et al. [11] noted that 46% of psoriasis and/or PsA cases seen in their multidisciplinary rheum‐derm clinic received a revised diagnosis, while a more recent prospective study by Ahnen et al. [10] reported that 15.2% of psoriasis patients with musculoskeletal symptoms referred to their interdisciplinary rheumatological‐dermatological clinic had their prior diagnosis changed. Although these studies report diagnosis changes during the combined clinic visit, none have examined the characteristics of the revised diagnosis. The goal of this study is to examine psoriasis patients seen at our tertiary institution's combined rheum‐derm clinic between November 2019 to March 2022 for a concern for PsA, specifically examining the revised diagnosis and the joint symptom similarities to psoriatic arthritis.

## 2 Material and Methods

A retrospective analysis was performed for patients seen between November 2019 and March 2022 at the combined rheumatology–dermatology clinic at our tertiary institution. The study was reviewed and approved by the institutional review board at our institution. Chart review was performed by the first author. Data was obtained from each patient visit documentation by the rheumatologist, dermatologist, or referring physicians. Joint symptoms data were obtained from the referral visit documentation, including referral letters and pre‐clinic notes, as well as the patient's history recorded at their RDC visit. Of note, for our analysis, small joints included DIP, PIP, MCP and wrist joints. Mechanical pain was defined as joint or musculoskeletal discomfort that worsens with physical activity and improves with rest, often resulting from structural or degenerative causes such as osteoarthritis, tendinopathy, or overuse syndromes. Chi‐square (*χ* ^2^) analysis was performed to compare the joint symptoms reported by patients at the referral visit. Significance level was set at 0.05.

PsA diagnoses were made by the evaluating rheumatologists during the RDC visit. Although the CASPAR criteria were not formally documented in the record for each patient, elements consistent with CASPAR, including current psoriasis, nail changes, dactylitis and negative rheumatoid factor, were considered in the clinical assessment. Serologic markers, including RF, ANA, ESR, CRP, HLA‐B27, CCP, Anti‐CCP and Vit D, were systematically reviewed and are explicitly summarised in Table S1. Due to variability in documentation across providers, formalised joint counts, enthesitis indices and skin severity scores were not uniformly available for analysis in this retrospective study. However, we captured joint symptom descriptors and skin findings (e.g., BSA involvement, nail changes) to approximate disease patterns. Current and prior medications, including biologics (adalimumab, secukinumab, ixekizumab, risankizumab), non‐biologic DMARDs (methotrexate, leflunomide, sulfasalazine) and topical therapies (e.g., clobetasol, Lidex), were extracted from clinical documentation and are summarised in Table S2.

## 3 Results

Two hundred and forty‐two unique patients were seen between Feb 2020 and March 2023 at the combined rheum‐derm clinic (RDC). Thirty‐four (14%) of the 242 patients had a history of psoriasis with or without psoriatic arthritis (PsA) and were referred to the RDC for further evaluation and management. Of the 34 referred psoriasis patients, 17 (50%) were female. Patient ages ranged from 13 to 82 with an average age of 47.4. Twenty (58.8%) of the 34 referred patients received a PsA diagnosis at the RDC visit while the other 14 (41.2%) patients were diagnosed with a non‐inflammatory musculoskeletal (NIMS) condition.

Of the 14 patients with a diagnosis of NIMS, 14 (100%) patients had some form of mechanical pain including osteoarthritis, shoulder impingement, tendinitis/tendinosis, patellofemoral syndrome, joint hypermobility syndrome, post‐traumatic arthritis, plantar fasciitis, or fibromyalgia. While fibromyalgia is not traditionally categorised as mechanical, we included it within the NIMS classification as it represents a non‐inflammatory cause of chronic joint and soft tissue pain. These diagnoses are summarised in Table 1. During the initial referral visit, 85% of PsA patients reported stiffness and/or swelling when compared to 35% of NIMS patients (*p* = 0.027). 75% of PsA patients reported small joint symptoms when compared to 51.7% of NIMS patients (*p* = 0.273) (Table 2 and Table 3).

**TABLE 1:** Non‐inflammatory musculoskeletal (NIMS) group (non‐PsA): Referral visit diagnosis versus combined rheum‐derm visit diagnosis.

Patient group	Referral visit diagnosis	Final diagnosis	Referring provider group
PsA	PsO and PsA	PsA, PsO	Rheumatology
PsA	PsO and suspected PsA	PsO and axial/peripheral PsA	Dermatology
PsA	PsO and suspected PsA	PsA, PsO, previous OA diagnosis	Dermatology
PsA	PsO and PsA	PsA and PsO	Rheumatology
PsA	PsA and PsO	PsA, PsO	Rheumatology
PsA	Possible PsA and PsO	PsA and PsO	Dermatology Physician Assistant
PsA	PsO and PsA	PsA and PsO	Dermatology
PsA	PsO and arthralgia of R knee and ankle	PsO, PsA	Dermatology
PsA	PsO and PsA	PsO and PsA	Rheumatology
PsA	PsO, possible peripheral PsA vs. seronegative RA	Peripheral PsA, dermatitis (chronic), seborrheic keratosis, inflammatory arthritis, seronegative RA, or CPPD	Rheumatology
PsA	PsO and PsA	PsO and PsA	Dermatology
PsA	PsO	PsO, PsA, Grover's disease, OA, axial spondyloarthritis	Dermatology
PsA	PsO vulgaris and possible PsA	PsO and PsA	Dermatology
PsA	PsA, PsO, hyperkeratotic dermatosis	Palmoplantar PsO, PsA	Dermatology Physician Assistant
PsA	PsO and PsA	PsO and PsA	Rheumatology
PsA	PsO and PsA	PsO and PsA	Dermatology
PsA	PsA and intertrigo vs. inverse psoriasis	PsO, PsA, atopic dermititis	Rheumatology
PsA	Rash and arthralgia	PsO and PsA	Family Medicine Nurse Practitioner
PsA	PsA and PsO	PsO and PsA	Rheumatology
PsA	PsO	PsO, PsA, OA, trochanteric bursitis	Dermatology
NIMS	PsO	Osteoarthritis	Dermatology
NIMS	PsO vulgaris	Mechanical pain	Dermatology
NIMS	PsO and PsA	Fibromyalgia, myofascial and mechanical pain	Dermatology
NIMS	PsO vulgaris	Mechanical back pain with radiculitis and mild shoulder impingement	Dermatology
NIMS	PsO with possible PsA	Patellofemoral syndrome or a periarticular soft‐tissue condition either tendinitis or sprain	Dermatology
NIMS	PsO, PsA	Regional osteoarthritis and a superimposed myofascial overlay/fibromyalgia	Dermatology
NIMS	PsO	Joint hypermobility/polyarthralgia	Dermatology
NIMS	PsO and arthralgia (unspecified joint)	Mechanical pain and post‐traumatic right wrist arthritis	Dermatology
NIMS	PsO and possible PsA	Joint hypermobility syndrome, regional osteoarthritis	Dermatology
NIMS	PsO and possible PsA	Regional osteoarthritis, shoulder impingement/left biceps tendonitis, left gluteus medius tendinosis	Dermatology
NIMS	PsO and possible PsA	Mechanical knee pain	Dermatology
NIMS	PsO and possible PsA	Plantar fasciitis or mechanical pain	Internal Medicine
NIMS	PsO and joint symptoms	Joint hypermobility syndrome	Dermatology
NIMS	PsO and arthritis	Osteoarthritis	Dermatology Physician Assistant

**TABLE 2:** Number of joint symptoms between psoriatic arthritis (PsA) and non‐inflammatory musculoskeletal (NIMS) groups.

	Stiffness/swelling	Bilateral	Axial	Large	Small	
PsA	17	13	4	7	15	20 patients
NIMS	5	12	6	11	8	14 patients

**TABLE 3:** *p*‐Values: chi‐square (*χ* ^2^) analysis comparing joint symptoms between psoriatic arthritis (PsA) and non‐inflammatory musculoskeletal (NIMS) groups.

Symptoms	*p* *
Stiffness and swelling	*p* = 0.027
Bilateral	*p* = 0.178
Axial	*p* = 0.15
Large	*p* = 0.12
Small	*p* = 0.273

* Significant level: *p* < 0.05.

**FIGURE 1:**
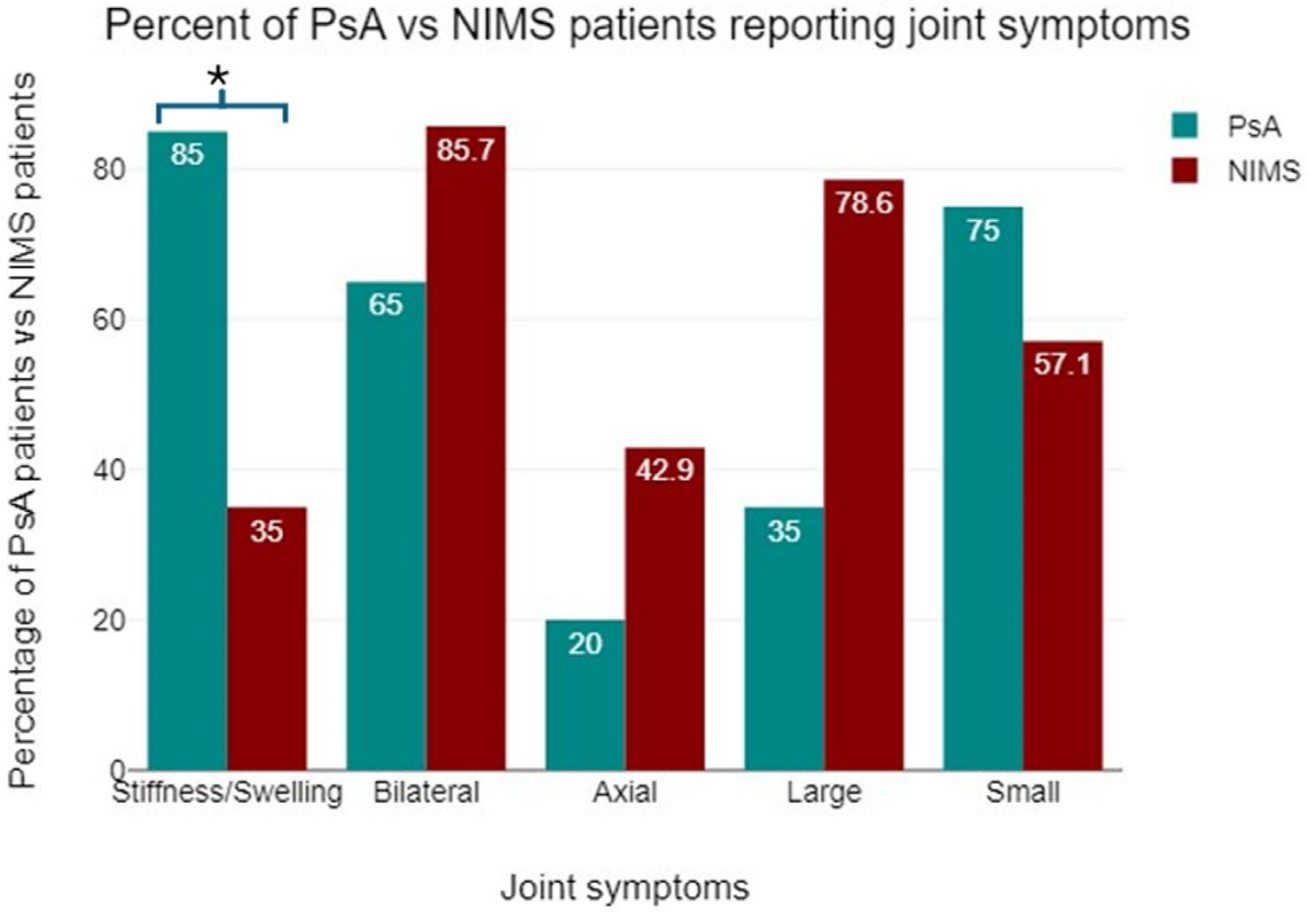
Percent of PsA versus NIMS reporting joint symptoms.

Conversely, 85.7% of NIMS patients reported bilateral joint symptoms when compared to 65% of PsA patients (*p* = 0.178). 42.9% of NIMS patients reported axial symptoms when compared to 20% of PsA patients (*p* = 0.15). 78.6% of NIMS patients reported large joint symptoms when compared to 35% of PsA patients (*p* = 0.12) (Table 2 and Table 3). Of these symptoms, only stiffness and/or swelling showed statistical significance (Table 3 and Figure 1). When available, laboratory testing (CRP, ESR, HLA‐B27, ANA) was reviewed, and patterns of elevation were more frequent in PsA than NIMS patients (Table S1). Among the PsA group, ESR and CRP were mildly elevated (ESR ranged from 3 to 35 mm/h and CRP ranged from 0.3 to 8.4 mg/dL) consistent with inflammatory disease activity. These markers were generally not obtained or reported for the NIMS group, reflecting the clinical judgement that inflammatory labs were not indicated for patients with non‐inflammatory diagnoses. Additionally, the available lab result data were too small for formal statistical analysis.

Among PsA patients, treatments included biologics (such as adalimumab, secukinumab, ixekizumab, risankizumab) and non‐biologic DMARDs (methotrexate, leflunomide, sulfasalazine). NIMS patients typically received conservative therapies, including topical agents, NSAIDs (naproxen) and analgesics (Tylenol) as treatment for their joint symptoms. This contrast in treatment further highlights the diagnostic distinction between inflammatory and non‐inflammatory musculoskeletal conditions. Referring physician data (Table 1) revealed that among patients who were ultimately diagnosed with non‐inflammatory musculoskeletal (NIMS) conditions, the majority were referred by dermatologists (10 of 12), with additional referrals from a dermatology physician assistant (1) and an internal medicine provider (1). These findings support our recommendation that targeted educational interventions be directed primarily toward dermatology providers and affiliated staff.

## 4 Discussion

In this study, we examined psoriatic patients referred to the rheum‐derm clinic at our tertiary institution between 2019 and 2022 to determine what types of arthritis are most commonly referred to this clinic. Among the psoriatic patients referred for PsA management, 20 (58.8%) received a final diagnosis of PsA while another 14 (41.2%) patients were diagnosed with a non‐inflammatory musculoskeletal (NIMS) condition. All the patients categorised here as NIMS had an associated mechanical pain component including osteoarthritis, shoulder impingement, tendinitis/tendinosis, patellofemoral syndrome, joint hypermobility syndrome, post‐traumatic arthritis and plantar fasciitis as noted in Table 1. Mechanical pain shares many features with psoriatic arthritis (PsA) which can complicate PsA recognition by physicians. This can lead to improper referral patterns that contribute to resource misallocation, delayed diagnosis, increasing healthcare costs and decreasing patient satisfaction.

Joint symptoms reported by psoriatic patients in this clinic included stiffness and/or swelling (22 of 34 patients), bilateral involvement (25 of 34 patients), small joint involvement (23 of 34 patients), large joint involvement (18 of 34 patients) and axial involvement (10 of 34 patients) as seen on Table 2. A significant difference was noted with stiffness and/or swelling symptoms, while other joint symptoms showed no statistically significant difference between both groups (Table 3 and Figure 1). The difference seen with stiffness and swelling is in line with the inflammatory symptoms expected for conditions like PsA. Additionally, previous studies have shown that symptoms such as joint pain with insidious onset, pain worse at night and/or at rest and pain improved with exercise can help differentiate inflammatory joint symptoms from mechanical pain [12]. We initially hypothesised that patients diagnosed with PsA would have a greater involvement of small joints when compared to NIMS patients, since inflammatory joint conditions often involve smaller joints. Our analysis revealed that the majority (75%) of PsA patients did in fact present with small joint symptoms; however, there was no significant difference between the PsA and NIMS groups (Table 3 and Figure 1). One likely explanation for this indifference is that osteoarthritis, a non‐inflammatory joint condition which typically also presents with small joint involvement, played a role in increasing the frequency of the small joint symptoms seen in the NIMS group.

Potential limitations of this study include small sample size and the retrospective nature of the study design. A tertiary referral center likely has a patient population that may be less generalisable to a non‐academic setting. Additionally, joint counts, enthesitis scores and standardised skin severity indices were not consistently recorded across all patients, and validated screening tools such as PEST or PASE were not used at the time of data collection, which could limit standardisation of symptom quantification. In summary, this report shows that over 40% of psoriasis patients referred to our combined clinic for psoriatic arthritis are ultimately diagnosed with a non‐PsA condition. When deciding to refer psoriasis patients for PsA evaluation, special attention should be given to symptoms of joint inflammation, particularly stiffness and swelling. Furthermore, improving the accuracy of referrals—particularly from dermatologists and dermatology‐affiliated providers—through educational outreach and potential implementation of structured screening tools may enhance diagnostic efficiency and optimise use of multidisciplinary clinics.

## Author Contributions

**Ugochukwu Kingsley Odega:** data curation, data analysis and writing – original draft preparation. **Adam Carlson:** supervision. **Wilson Omesiete and R. Hal Flowers:** conceptualisation, draft editing and supervision.

## Ethics Statement

The study was reviewed and approved by UVA IRB—HSR # 23900.

## Consent

The patients in this manuscript, where applicable, have given written informed consent to publication of their case details.

## Conflicts of Interest

R. Hal Flowers has served as a principal investigator for Abbvie, Acelyrin, Clinuvel, Regeneron/Sanofi and Sun Pharmaceuticals. He has served on advisory boards for Argenx, Bristol‐Myers Squibb and Janssen.

## Supporting information


**Data S1:** ajd14585‐sup‐0001‐supinfo.rtf.

## Data Availability

The data that support the findings of this study are available on request from the corresponding author. The data are not publicly available due to privacy or ethical restrictions.
