# Supplementary material for: Characterising Alternative Diagnoses to Psoriatic Arthritis in a Rheumatology‐Dermatology Combined Clinic
Source: Australas J Dermatol. 2025 Aug 21;66(8):462–7. doi: 10.1111/ajd.14585 (PMC12687689; doi:10.1111/ajd.14585)
Supplement: Supplementary file 1 — Data S1: ajd14585‐sup‐0001‐supinfo.rtf. [file AJD-66-462-s001.rtf]

Table S1. Combined available lab values for PsA and NIMS groups

Patient group	RF	ANA	ESR	HLA-B27	Vit D	CCP	AntiCCP	CRP	
PsA	<13					<0.5			
PsA									
PsA			30					0.7	
PsA			35					0.3	
PsA			3					0.6	
PsA	<13	positive(1:160)	35			<0.5		0.3	
PsA									
PsA									
PsA			12					0.6	
PsA			3					<0.1	
PsA			24					0.9	
PsA									
PsA									
PsA									
PsA								8.4	
PsA									
PsA								1.7	
PsA	<13	negative	26						
PsA									
PsA									
NIMS									
NIMS									
NIMS									
NIMS									
NIMS									
NIMS									
NIMS									
NIMS									
NIMS									
NIMS									
NIMS									
NIMS	10.8	negative		negative			16		
NIMS									
NIMS									

Table S2 . Skin and Joint symptoms treatment for PsA and NIMS groups


Patient Group	Past Skin Treatment	Skin treatment at RDC visit presentation	Skin Improvement	Past Joint Treatment	Joint treaatment at RDC presentation	Joint symptom Improvement	RDC Treatment Recommendation	Final Diagnosis	
PsA	sulfasalazine, oral and SQ methotrexate, secukinumab, etanercept(allergic), adalimumab and guselkumab 	clobetasol, leflunomide	no	none	Leflunomide 	yes	continue Taltz and leflunomide, clobetasol, switch to ketoconazole and begin triamcinolone. begin iodochlorhydroxyquin 3%-LCD 5%-triamcinolone 0.1% EQ Cr/Ung	PsA, PsO	
PsA	none	topical Lidex solution for scalp, and triamcinolone for elbow. Betamethasone and calciprotriene 	yes	none	naproxene	yes	continue topicals, restarted naproxen	PsO and Axial/Peripheral PsA	
PsA	adalimumab, secukinumab	Risankizumab and Enstilar	yes	none	Risankizumab and tylenol	no- Risankizumab but transient relieve for tylenol	start methotrexate, continue risankizumab and enstilar	PsA, PsO, previous OA diagnosis	
PsA	 taltz	neosporin	no	etanercerpt, adalimumab, secukinumab	ixekizumab	yes	continue ixekizumab and clobetasol 	PsA and PsO	
PsA	adalimumab(caused worsening inverse psoriasis), methotrexate(caused GI upset), greer's goo, desonide 	secukinumab, clobetasol, tacrolimus	yes- however certain sites of the skin disease is refractory 	adalimumab(caused worsening inverse psoriasis), methotrexate(caused GI upset)	seckinumab, ibuprofen	yes	continue secukinumab, clobetasol,t-gel shampoo, tacrolimus, ketoconazole. begin dovonex, mupirocin	PsA, PsO	
PsA	triamcinolone, clobetasol	selenium sulfide, hydrocortisone	yes- initially but began to worsen over time	none	diclofenac 	yes	start Elidel continue triamcinolone, clobetasol, vaseline. PsA- meloxicam 	PsA and PsO	
PsA	methotrexate(did not respond), adalimumab(lost efficacy), topicals	none	yes- but lost effectiveness 	none	none		continue secukinumab, restart triamcinolone ointment, add calcipotriene	PsA and PsO	
PsA	t-gel shampoo, ketoconazole 2% shampoo, tea tree oil	hydrocortisone 	yes	none	none		PsO-continue clobetasol for scalp, and hydrocortison for penis, PsA- start naproxen 	PsO, PsA	
PsA	Intralesional triamcinolone	apremilast, clobetasol, Tgel shampoo	no	none	apremilast	no	start adalimumab and continue clobetasol, discontinue otezla	PsO and PsA	
PsA	none	none		none	none		clobetasol, tofacitinib	 peripheral PsA, Dermatitis(chronic), Seborrheic keratosis, Inflammatory arthritis, seronegative RA, or CPPD	
PsA	acitretin, methotrexate, adalimumab, infliximab, enbrel	vasoline petrolatum 	infliximab worked but d/c because she moved	methotrexate, adalimumab, infliximab(remicade), enbrel	none		restart Infliximab, continue triamcinolone(was started on referral visit) 	PsO and PsA	
PsA	metotrexate, topical steroids, Wilson's Wonder (LCD 8%, salicylic acid 4%, and triamcinolone 0.025% ointment) 	none		metotrexate	leflunamide and tylenol	yes	clobetasol for PsO, trial of celebrex, continue tylenol and leflunomide. 	PsO, PsA, Grover's disease, OA, Axial Spondyloarthritis	
PsA	clobetasol, Wilson's wonder, combination iodochlorhydroxyquin 3%-LCD 5%-triamcinolone 0.1%, betamethasone ointment, Dovonex, methotrexate, adalimumab, Triamcinolone 	ustekinumab	yes	methotrexate, adalimumab 	ustekinumab	no	transition from stelera to cosentyx	PsO and PsA	
PsA	clobetasol ointment, calcipotriene cream, urea cream, methotrexate, adalimumab, etanercept, and secukinumab and ustekinumab, leflunomide, prednisone 	acitretin 	yes	methotrexate, adalimumab, etanercept, secukinumab, allopurinol, leflunomide, ustekinumab	ustekinumab	yes	continue acitretin, continue ustekinumab injections and resume leflunomide	Palmoplantar PsO, PsA	
PsA	Methotrexate	adalimumab, cyclosporine, clobetasol ointment, hydrocortisone 2.5% ointment	yes	methotrexate	adalimumab	yes	Continue adalimumab, Continue cyclosporine, continue clobetasol ointment, Continue hydrocortisone, Consider addition of calcipotriene cream 	PsO and PsA	
PsA	etanercept	clobetasol ointment, calcipotriene cream, and triamcinolone 0.025% cream, ketoconazole shampoo 	no- eternercept, helped but discontinued prior to transplant 	none	none		Continue clobetasol solution, change shampoo to MG217 tar shampoo, continue clobetasol ointment and dovonex cream. Intraarticular steroid injections	PsO and PsA	
PsA	none	adalimumab and methotrexate 	yes	none	adalimumab and methotrexate 	yes	continue mathrotrxate and adalimumab, Trial of Greer's goo as well as pimecrolimus cream,  	PsO, PsA, atopic dermititis 	
PsA	cortizone, benadryl	none		none	none		discontinue triamcinolone and start topical clobetasol 0.05% ointment, start topical calcipotriene 0.005% cream, restart meloxicam(advised to take take freqently) 	PsO and PsA	
PsA	none	humira	no	 etanercept, ustekinumab, secukinumab, golimumab, and methotrexate	humira	no	trial of guselkumab(Tremfya), clobetasol ointment and calcipotriene cream 	PsO and PsA	
PsA	none	humira	no	none	humira	no	trial of Tremfya, clobetasol ointment, and triamcinolone 0.1% ointment. Start celebrex	PsO, PsA, OA,trochanteric bursitis	
NIMS	methotrexate, triamcinolone, tacrolimus, Eucrisa	humira, naproxen,dovonex	yes	methotrexate	naproxen	yes	continue humira,naproxen,dovonex, add duloxetine 	Osteoarthritis 	
NIMS	none	clobetasol, triamcinolone	no	none	aleeve 	yes	start stelara for skin and continue aleeve	Mechanical pain	
NIMS	methotrexate, adalimumab(Humira),infliximab(Remicade), secukinumab (cosentyx)	Inflixmab (inflectra)	infliximab-no, reports remicade helped but lost insurance coverage	methotrexate, Humira, Remicade, and Cosentyx	inflectra	no	clobetasol ointment and dovonex cream for skin. stop infliximab, uptritrate Gapentin for pain 	Fibromyalgia, Myofascial and Mechanical pain	
NIMS	Stelara, UV light therapy	Humira	yes	Mobic,metotrexate	naproxen, humira	no for humira, trainsent-aleve	continue humira, discontinue aleve, start celebrex and tylenol	Mechanical back pain with radiculitis and mild shoulder impingement	
NIMS	clobetasol, betamethasone, triamcinolone, Lidex, Dovonex  	 betamethasone	yes	none	none		continue topical corticosteroids use as needed. Tylenol and Aleve as needed for joint	Patellofemoral syndrome or a periarticular soft-tissue condition either tendinitis or sprain	
NIMS	T-gel shampoo, significant tanning bed use for PsO per patient	apremilast, topical clobetasol ointment and calcipotriene 	yes	none	none		continue apremilast as well as topical clobetasol ointment and calcipotriene as needed. Tylenol and naproxen as needed for joint. Regular exercise/physical activity for joint.	Regional osteoarthritis and a superimposed myofascial overlay/fibromyalgia	
NIMS		adalimumab	yes	none	none		Continue adalimumab for skin	Joint hypermobility / Polyarthralgia	
NIMS	etanercept	calcipotriene, betamethasone, tacrolimus, light therapy(tanning bed) 	yes	none	celebrex and tumeric supplements	yes	Start adalimumab for skin, continue topical clobetasol and calcipotriene ointment, continue celebrex 	Mechanical pain and post-traumatic right Wrist Arthritis	
NIMS	adalimumab	ixekizumab and prednisone	yes	none	none		continue ixekizumab 	Joint hypermobility syndrome, regional Osteoarthritis	
NIMS	methotrexate and NB-UVB 	clobetasol and lidex	no	none	Ibuprofen and Tylenol	yes	start adalimumab for skin, continue NSAIDs and Tylenol as needed 	Regional Osteoarthritis, Shoulder Impingement / Left Biceps Tendonitis,Left Gluteus Medius Tendinosis	
NIMS	NBUVB	betamethasone and tacrolimus ointment	no	none	none		continue betamethasone and tacrolimus ointment. Trial Tylenol, restart Naproxen 	Mechanical knee pain	
NIMS		clobetasol ointment, calcipotriene cream, mometasone cream	yes	none	intralesional triamcinolone injection	yes but transient	Continue clobetasol ointment, continue calcipotriene cream, mometasone cream, intralesional triamcinolone injection	Plantar fasciitis or mechanical pain. 	
NIMS	methotrexate	clobetasol, calcipotriene cream, home phototherapy  	yes	none	none		continue phototherapy, continue topical clobetasol ointment and calcipotriene cream. tylenol and naproxen as needed for joints.  	Joint hypermobility syndrome	
NIMS		clobetasol foam, fluocinolone 0.01% oil, and triamcinolone 0.1% ointment, calcipotriene 	yes 	none	none		continue clobetasol, antiseborrheic shampoos, Start tylenol for arthritis and voltaren gel.	Osteoarthritis	
